# Bioactive Compounds from Organic Waste

**DOI:** 10.3390/molecules29102243

**Published:** 2024-05-10

**Authors:** Benito Parra-Pacheco, Byanka A. Cruz-Moreno, Humberto Aguirre-Becerra, Juan Fernando García-Trejo, Ana Angélica Feregrino-Pérez

**Affiliations:** Research and Postgraduate Division, School of Engineering, Universidad Autónoma de Querétaro, Campus Amazcala, Carretera a Chichimequillas Km 1 s/n, Amazcala, El Marqués 76265, Querétaro, Mexico; benito.parra@uaq.mx (B.P.-P.); mibyvis14@gmail.com (B.A.C.-M.); humbertoagbe@hotmail.com (H.A.-B.)

**Keywords:** organic waste, bioactive compounds, biological properties, added value, waste revaluation

## Abstract

The reuse and reincorporation of waste are the principles of circular economies. Compost, biofuels, animal feed, dyes, and bioactive compounds can be obtained from the revaluation of organic waste. Research on this subject is scarce and limited to specific sectors, such as agriculture and agroindustry, leaving aside others that generate large quantities of organic waste, such as floriculture. The remains of these sectors have a low decomposition rate compared to other organic wastes. They are a source of bioactive compounds (e.g., essential oils, pigments, phenols) that can be reincorporated into the production chain of various industries. This review describes the composition of waste from agroindustry, agriculture, and floriculture, analyzing their potential revalorization as a source of bioactive compounds and an alternative supply source.

## 1. Introduction

**Figure 1:**
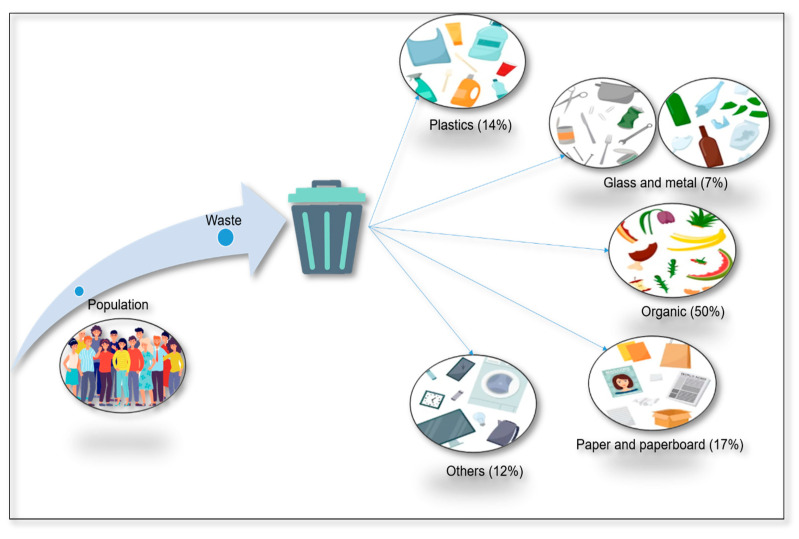
Representative scheme and percentages of waste generation.

Population growth causes greater demand for health services, housing, and food, resulting in a high amount of residues that are generally classified as inorganic and organic [1] (Figure 1). Globally, more than 2.1 billion tons of garbage is produced each year and is expected to increase by 70% by 2050 [2]. This situation has caused social, economic, health, and environmental concerns. In this sense, agriculture, livestock, aquaculture, the food industry, and other agroindustrial sectors generate large amounts of organic residues such as fruits, vegetables, leaves, stems, foliage, husks, seeds, pulp, stubble, bagasse, husks, straws, manure, feathers, whey, and other animal byproducts [3,4,5]. Other areas not directly related to the agroindustry (i.e., gardening) generate leaves, branches, flowers, soil, and insects as waste [6]. Organic garbage is generally mixed, making its composition highly variable. Separation methods are misused, leading to inadequate transformation and low separation recurrence, resulting in environmental problems such as erosion, the release of greenhouse gases (e.g., CO_2_), and air, soil, and aquifer pollution [7,8]. On the other hand, organic matter contains a high number of sugars [9], lipids [10], proteins [11], lignocellulosic biomass, and other functionalized molecules [12,13]. The extraction of these compounds would allow for their revaluation, reducing contamination and infection sources. This topic has captured the scientific community’s attention as many works have reported using organic waste for different applications. For example, rice husk contains amorphous silica, which can be used to manufacture non-structural concrete blocks [14] and masonry bricks [15]. Moreover, the interaction of microorganisms with residual biomass in saccharification and fermentation techniques allows for the obtention of lactic acid [16,17], succinic acid [18], and polyhydroxy butyrate [19], which are used in the synthesis of bioplastics.

Research has focused on organic waste conversion processes to obtain different byproducts. Different phenolic compounds and pigments (e.g., chlorophylls, anthocyanin, betalains, and carotenoids) have been extracted from fruit and vegetable trash (e.g., seeds, peels, or pomace from grapes, tomatoes, and red beet) [20]. These compounds can be used as food additives, natural colorants, and color intensifiers [21]. Additionally, dietary fiber, phenolic compounds, flavoring agents, aromas, enzymes, and organic acids can be obtained from mango, banana, grape, potato, tomato, garlic, lemon, orange, and carrot residues [22]. Pectin is another biocompound that can be extracted from coffee mucilage and citrus trash [23,24]. Furthermore, bioplastics can be obtained from organic waste to produce biofilms for plant fertilizers, intelligent packing, biomedical devices, and sensors [25].

The absorbent properties of waste can be used to extract colorants from wastewater; for example, rice husks can absorb pigments from the textile industry, avoiding contamination problems [26,27]. Organic residues can be used as compost to enrich agricultural soil [24], substituting chemical fertilizers [25]. On the other hand, implementing organic waste as animal food supplementation in different species has been studied [28] and proposed as a cheaper alternative with optimal nutritional values in food production for pigs, birds, ruminants [29], and fish [30]. Furthermore, biofuel production is one of the most exploited applications for lignocellulose waste revaluing [31], representing an alternative to the increasing cost and low availability of non-renewable fossil fuels [32].

**Figure 2:**
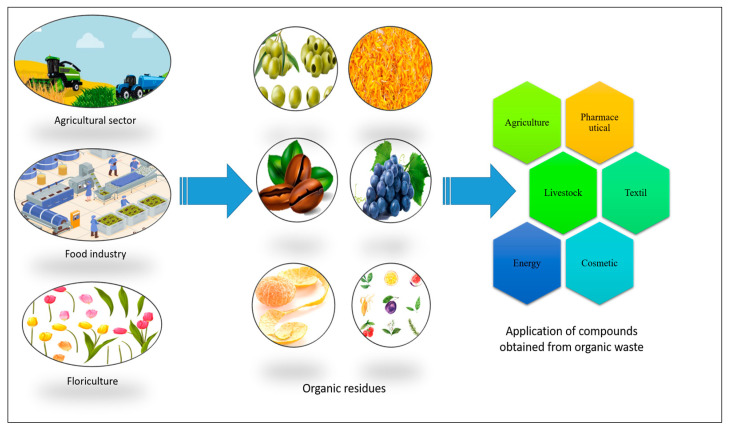
Applications of biocompounds from organic waste generated in different sectors.

Agricultural residues are the most studied for revaluing purposes, whereas floriculture garbage has been less explored. Worldwide, the Netherlands, Germany, France, Spain, and Italy lead flower production. In 2019, the Netherlands reported 34.3 thousand hectares for producing ornamental flowers [33], followed by Poland with 8.77, France with 8.74, Italy with 8.31, and the United Kingdom with 7.00 [34]. Analogously, India produces 700 million tons of flower waste annually. In some countries, such as India and Sri Lanka, annual flower production losses are nearly 40%, and in some cities, such as Aurangabad, floral waste is 50kg per day [35]. The Indian cities exhibiting the significant production of this residue are Varanasi and Surat, with 10 to 1.5 tons per day [36]. In different temples in Chennai city, waste generation ranges from 125 to 800 kg per day [37]. Another study estimates that flower waste in India is approximately 4738 tons per day [38], and 50 tons of tulip petals in Turkey annually [39]. These residues exhibit excellent potential for incorporation into other processes [40]; unfortunately, this waste is not treated, generating contamination problems [41]. However, they can be utilized to produce handmade paper and contain compounds such as essential oils, pigments, and phenols, which can be used in pharmaceutical, food, biofuel, and cosmetic industries [37,42] (Figure 2).

Research on organic waste’s potential in different sectors has recently emphasized biocompound extraction methods for their incorporation into other production processes. This work aims to present a compilation and analysis of bioactive compounds in organic waste and their possible use in other industrial applications. This review focuses on residues derived from the food industry (Section 3), crops (Section 4), and other sources (e.g., floriculture) and their possible applications (Section 5).

## 2. Methodology

The articles in this review were found from different academic publishers such as MDPI, Springer, Science Direct, Academic Google, Taylor and Francis, and Research Gate. Keywords such as bioactive compounds, pigments, flavonoids, phenols, fatty acids, fibers, carbohydrates, proteins, and essential oils from the organic waste of agroindustry, food industry, agriculture, and floriculture were used to filter the most important information. Databases were visited to obtain information on flower-producing countries and residues containing bioactive compounds. The information was synthesized and classified according to the date and main compounds of residues of agriculture, agroindustry, food industry, and flowers. The discrimination criteria for the writing were created as follows: the review of compounds in organic waste was performed with articles and databases between the years 2002 and 2023. For the content of Table 1, which establishes extraction methods, references from articles and book chapters between the years 2012 and 2023 were used. For Table 2, on specific compounds from organic waste, references from 2017 to 2023 were used.

## 3. Waste Derived from Food Industry

**Figure 3:**
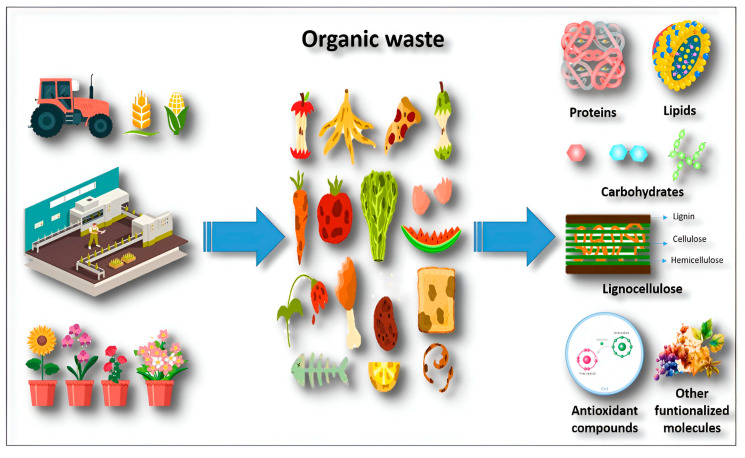
Specific biocompounds contained in organic waste from different agroindustrial sectors such as agriculture, the food industry, and floriculture.

Food processing generates large amounts and a wide variety of organic waste (Figure 3). For example, olive fruit is a traditional Mediterranean cultivation, with 90% of the world’s oil and table olives produced for human consumption [43]. In 2019, olive production was headed by Spain (5,965,080.00 tons), followed by Italy (2,194,110.00 tons), Morocco (1,912,238.00 tons), Turkey (1,525,000.00 tons), and Greece (1,228,130.00 tons) [44]. Olives and olive oil contain different types of lipids (unsaturated, monounsaturated, and polyunsaturated fatty acids) and other compounds, in fewer concentrations, that benefit human health, such as phenols, sterols, squalene, tocopherol, proteins, and pigments [45]. Grapes have the same importance as olives in the Mediterranean, since they are used for producing different types of wine with different varieties of grapes rich in phenolic acids, flavonoids, tannins, and anthocyanins, providing excellent flavor and aromas [46].

Researchers have described extracting and removing polyphenolic compounds and natural dyes from olive oil and wine industry residues, including those from the production process and cultivation (i.e., leaves and fruits) [47]. An estimated three tons of pruning waste per cultivated hectare of olive trees are produced annually, representing a cheap and unexploited source of bioactive compounds [48]. Amurca, orujo, and alpeorujo constitute the olive pomace, rich in phenolic compounds such as oleuropein, which is most abundant in olive leaves, and hydroxytyrosol, the most bioactive phenol in olives [49]. These phenols have been attributed with antioxidant [50], antiviral [51], antimicrobial [52], and antibacterial properties [53]. Additionally, terpenes [54], carotenoids [55], tocopherols [56], phytosterols [57], and phytoene with long chains of alcohols have been identified in olive pomace [58]. Thus, this residue has desirable bioactive compounds for the food, pharmaceutical, and cosmetic industries. The residues of wine, such as seed, fruit, pulp, and leaves, contain polyphenols and antioxidants [59]. Proanthocyanidin in red grapes provides benefits against human diseases such as inflammation, cardiovascular problems, diabetes, hypertension, and microbial infections [60]. Vitamin A, vitamin E, and carotenoids such as alpha-carotene, beta-carotene, xanthine, beta-cryptoxanthin, and lycopene are nutraceuticals with antioxidant properties [61].

Citrus fruit processing generates a large amount of organic garbage (e.g., peels, seeds, and pomace) used to feed animals or disposed of in landfills. However, this residue is a source of raw materials with various applications in the food and non-food sectors [62]. After processing, different variants of pectin are extracted from orange, mandarin, bitter orange, lime, lemon, and grapefruit. Pectin could be applied as food biofilm, emulsifier, texture modifier, thickener, and for soft drinks, fruit beverages, dairy products, confectionery, and bakery fillings [63].

The wine industry is one of the largest worldwide. Spain is the leading wine producer in Europe, with an average of 1.1 million hectares used for grapevine production [47]. In this industry, two indirect residues are not used: grapevine shoots [64] and leaves [65]. Cellulose, hemicellulose, lignin, and bioactive compounds such as tannins, phenols, lactic acid, volatile compounds, and some dyes can be obtained from grapevine shoots. The grapevine leaves are used based on their maturity. For example, in Turkey, they are used in their green form as part of gastronomy and folk medicine as they are considered healthy and nutritious [66]. Senescent leaves have not been thoroughly studied, although they are deemed to have flavonoids, anthocyanins, and carotenoids due to their color (yellow to brown), which makes them a potential source of dyes. Among the direct residues of wine production, the skin, seeds, and must (vinification lees) are a potential source of colorants, phenols, flavonoids, proanthocyanidins, condensed tannins, fatty acids, lactic acid, and tartaric acids, considering that the proportions and characteristics change depending on the grape variety [67].

Coffee agribusiness generates a large amount of waste. According to the International Coffee Organization, in December 2018, 10.43 million bags of coffee beans were exported, representing an increase of 0.9% compared to December 2017 [68]. Spent coffee grounds, husk, and coffee pulp are residues that can harm the environment [69]. The grains and spent coffee grounds have proteins, caffeine, oil, and carbohydrates such as mannose, galactose, glucose, and arabinose [70,71]. Moreover, chlorogenic acid and its derivatives have been reported in coffee, pulp, husk, and waste coffee byproducts. Given their antioxidant properties and dietary fiber, these compounds can be applied as a food additive or supplement with high nutritional value [72,73,74].

Another example is the cocoa industry. The International Cocoa Organization reports that in 2015, 3.9 million tons of cocoa beans were collected, with an estimated production of 16 million tons of residual biomass [75]. In 2020, as exporters, the Netherlands, Germany, Nigeria, Ghana, and France recorded an income of USD 24.48 million for this residual biomass composed of cocoa pods, husk, and pulp [76]. Cocoa is a natural product for snacks, beverages, and chocolates. During cocoa processing, the waste generation is estimated at 85% of the total production, constituted by cocoa bean husks, cocoa shells, and pulp [77]. Research points to two extraction routes to obtain bioactive compounds from the residual biomass: bioconversion and solvent extraction, highlighting those with antioxidant, antimicrobial, and antiviral capacities [78] or other compounds such as polyphenols [79], pectin, phytosterols, dietary fiber, with anti-inflammatory, anti-hypertensive, anti-diabetic and anti-hypercholesterolemic properties [80]. Additionally, cocoa shell has polysaccharides, lignin, and cellulose as a dietary fiber that could be used to prepare chocolate cookies and cakes with coloration and flavor advantages over commercial products [77].

Among the applications of biocompounds that can be used from organic waste, it is worth mentioning the extraction methods and their requirements, as well as the different methodologies to conserve their properties, decrease degradation, improve performance, reduce time and costs, and mainly maintain the structure and shape of the biocompounds to be extracted [81]. Several aspects should be considered during the scaling process, such as instrumentation, batch or continuous extraction, economic aspects, and energy and solvent consumption. Methods for extracting biocompounds are generally classified into two categories with advantages and disadvantages (Table 1).

**Table 1:** Classification, advantages, and disadvantages of extraction methods for biocompound extraction from organic waste.

	Method	Solvent	Advantage	Disadvantage	Author
Conventional extraction methods	Percolation	Water and Solvents	The equipment is simple and applicable to a wide range of organic matter.	Unstable components due to temperature. High solvent and energy consumption. Long extraction time.	[82]
	Maceration	Water and solvents	Easy-to-use material and implementation. Minor energy consumption (electricity).	It takes a long time to extract components (days to weeks). Significant consumption of solvent. Non-exhaustive extraction.	[83]
	Decoction	Water	Used for phenolic compounds. Easy to use.	Significant energy consumption. Heating takes minutes to hours. Not for thermolabile and volatile compounds.	[84]
	Soxhlet extraction	Solvents	Lower amount of solvent. Filtration after extraction is not required. Easy-to-use equipment.	Long time for extraction, and high volume of solvent.	[85]
	Hydrodistilation	Water	Volatile compounds extraction. Easy-to-use material and equipment.	Cannot be used for thermolabile compounds. Long extraction time. Possible chemical change.	[86]
Eco-Friendly/Green Extraction Method	Ultrasound-assisted extraction (UAE)	Solvents	Lower amounts of solvent, low energy consumption and extraction time, major extraction efficiency, preservation of bioactive compound stability, and widespread industrial applications.	Not recommended for thermolabile compounds. Heat generated during extraction can modify compound’s structure.	[87]
	Microwave-assisted extraction (MAE)	Solvents	Low-cost equipment that requires reduced extraction time and solvent quantity. Batch extraction.	More complicated and time-consuming than blending. High pressure.	[88]
	Supercritical fluid extraction (SFE)	Solvents, mainly carbon dioxide	High selectivity for non-polar compounds. Recommended for thermolabile compounds.	High cost and complex operation.	[89]
	Pressurized liquid extraction (PLE)	Solvents	Lower solvent consumption. Short processing time. Possibility to perform more extraction cycles and samples throughout.	High instrumentation cost and long cell preparation	[90]

## 4. Crop Residues

Crop residues are the inedible parts of plants left in the field after harvest, including those discarded in the transformation processes, such as the peel and seeds of fruits. Agricultural activity generates billions of metric tons of waste annually, which must be adequately managed and treated [91]. According to [92,93], cereals are the main crop worldwide, which produces a large amount of waste, such as straw, stem, and leaves that contain lignocellulose compounds [94], which are mainly used for bioenergy generation.

Some methodologies to obtain bioactive compounds from crop waste have been described [95,96,97,98], focusing on extracting those with high antioxidant capacity, given the great demand of the food, pharmaceutical, and cosmetic industries for natural products with this characteristic. Other techniques focus on obtaining aromatic [99] and phenolic [100] compounds from the coffee husk; obtaining polyphenols, carotenoids, nitrogenous compounds, and citric acid from orange peel [101,102]; and obtaining alternariol and tenuazonic acid, compounds with antibacterial activity, from sugarcane bagasse, corn, and wheat bran [103]. Additionally, numerous articles describe crop residues as a source of antioxidant compounds, mainly polyphenols, for potential implementation in food [104] and livestock feed [105]. In this line, investigators evaluated the leaves and stems of tomato, broccoli, watermelon, cucumber, and leek crops compared to the direct crop products, observing that the concentration of bioactive compounds, such as phenolic and carotenoids, depends on the type of crop and that, in some cases, the residues contain a higher concentration, compared with the fruit or vegetable. For example, tomato and watermelon leaves and stems have more lipophilic antioxidant-type compounds, such as carotenes, than fresh fruit, concluding that crop residues are an excellent source of bioactive compounds for food, cosmetics, and pharmaceutical applications.

The Food and Agriculture Organization (FAO) estimates that at least a third of the food produced worldwide is wasted annually, derived from the post-harvest, processing, distribution, and consumption chains [106]. Bioactive compounds such as carotenoids, phenolic compounds, fatty acids, tocopherols, and flavonoids can be obtained from fruit seeds and used in the pharmaceutical or alimentary sectors; therefore, developing new products or incorporating fruit waste is necessary and is increasing due to the circular economy trends and the full use of natural resources. In this sense, products containing fruit residues provide biological (through the incorporation of bioactive compounds) and economical (by including residues) added value [107]. For example, some studies have increased the antioxidant capacity of yogurt by adding grape skin flour and berry extract [108,109]. Other studies added apple and avocado peel phenolic compounds to oil to inhibit oxidation [110,111]. Different bioactive compounds from plants and fruits can be used as natural antioxidants to incorporate in meat processing [112] to preserve color, flavor, and nutritional quality when exposed to heat, oxygen, free radicals, light, and additives [113]. For example, citrus fibers are an alternative to sodium phosphate for meat curing [114]. Applying phenolic compounds from *Terminalia arjuna* to ground pork can form peroxide and thiobarbituric acid, preventing protein oxidation and rancid odor and color [115].

## 5. Flower Residues

**Figure 4:**
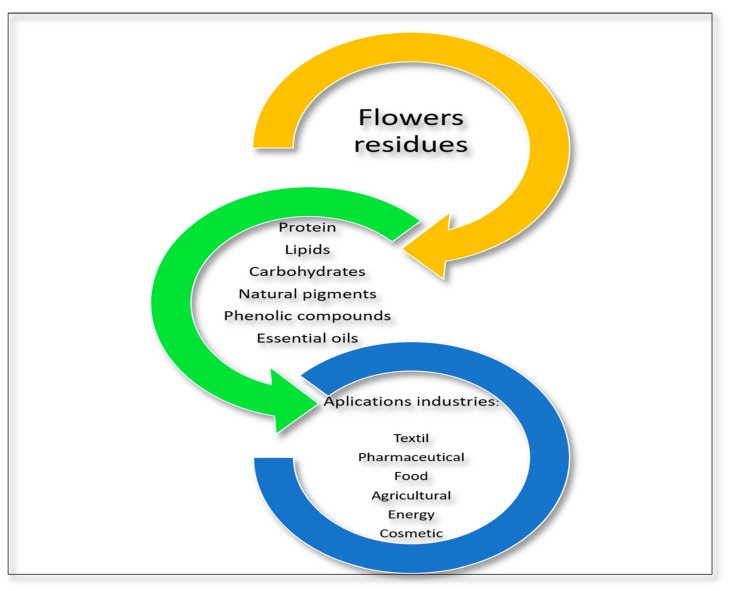
Compounds present in flower residues and possible industrial applications.

Floriculture has increased consistently in the last 20 years, with an annual growth of 6% to 9%. According to the International Association of Horticultural Producers, the Netherlands produces 52% of all flowers worldwide [116]. In contrast, India is one of the leading producers of floral waste, at an estimated 700 million tons annually [117]. Residues from this sector have diverse bioactive compounds; however, they are not considered within the previous classifications as they are not directly linked to food production processes, even though some flowers can be considered food in several countries. Flowers are used for various purposes, including gardening, decoration, gastronomy, and cultural and religious functions, where the latter are the primary sources of floral waste. Considering this residue’s decomposition is slower than other organic residues [117], the interest in studying their potential use as a source of bioactive compounds and incorporation in industrial processes has increased. Flowers contain proteins, carbohydrates, saturated and unsaturated lipids, carotenoids, flavonoids, organic acids, phenols, alkaloids, and terpenoids, as well as vitamins, minerals, and antioxidants [37,118,119], making them an attractive source of bioactive compounds (Figure 4). Additionally, flowers are used within medicinal practice given the therapeutic properties of their essential oils, water, and decoctions [120]. Moreover, flowers have many compounds (e.g., flavonoids, carotenoids, and anthocyanins) that grant them a wide range of colors and make them a potential source of natural pigments [121].

Antioxidant capacity and antimicrobial and antifungal properties have been attributed to flowers. For example, investigators have indicated a significant relationship between antioxidant power and the total content of phenolic compounds [122], and others have suggested that the antioxidant activity of flowers is due to flavonoids, phenolic acids, anthocyanins, and alkaloids [123]. Their general and individual quantification is essential for understanding the true bioactivity potential, considering that different species have different types and amounts of these compounds. Additionally, the extraction method influences the compound concentration. For example, subcritical water extraction was more efficient in releasing phenols than the conventional method in marigold (*Tagetes erecta* L.) flower debris [124].

Furthermore, flowers’ polyphenols, flavonoids, and tannins might cause antibacterial activity against some Gram-positive and Gram-negative bacteria [125]. *Madhuca latifolia* flowers could be a potential bioresource for producing exopolysaccharides with antibacterial, antifungal, and antiviral activity [126]. Volatile compounds, such as essential oils composed of terpenes, aliphatic hydrocarbons, aromatics, and alkanes, have been reported to exhibit antimicrobial and antifungal activity, the latter due to their hydrophobic nature [127]. In this sense, essential oils interact with the lipids of the bacterial cell membrane, increasing its permeability and leading to the leakage of ions and other cellular materials, inducing cell death. Other proposed mechanisms are the coagulation of the cytoplasm [128], membrane protein damage, ATP hydrolysis, decreased ATP synthesis [129], and the formation of voltage-dependent ion-permeable channels by biopeptides leading to microorganisms’ death due to an osmotic imbalance [130].

In medicine, value-added compounds derived from flower residues can be used. The essential oils of various flowers are used to treat several ailments. For example, passionflower (*Passiflora incarnata*) helps to decrease symptoms of stress and insomnia [131]; calendula (*Calendula officinalis*) extract is used in diverse medical conditions as a product leading to skin healing, de-inflammation, the production of granulation tissue, and the prevention of acute post-radiation dermatitis. [132]; chamomile (*Matricaria chamomilla* L. and *Chamaemelum nobile* L.) is employed in skin disorders and relieves muscular cramps [133]; rose oil (*Rosa* spp.) is utilized as a fragrance, and in cosmetics and food [134]; and arnica (*Arnica* spp.) oil is used in traditional and homeopathic medicine for its antiseptic, analgesic, and anti-inflammatory characteristics [135]. Acacia dealbata flowers were employed for their antibrowning, anti-lipogenic, and anti-inflammatory properties, and for their cytotoxicity on colon carcinoma HCT-116 and lung adenocarcinoma A549 cells [136]. The alternatives for the management of flower residues include obtaining syrup [37], incense [137], and rose (*Rosa indica*) water generation [138], among others (Table 2).

**Table 2:** Main biocompounds obtained from organic waste from different sectors including floriculture.

	Biocompounds	Organic Waste	Reference
Bioactive compounds from food industry and crops.	Lemon seed oil (DM) Yield (%): 26.65–35.85 Total polyphenol: 82.46–165.90 µg GAE/mL Flavonoids: 11.88–21.69 µg QE/mL	Lemon seed	[139]
	Total polyphenols in peel: 3.51–5.17 mg GAE g^−1^ (FW) Total phenol content in seed: 4.44 mg GAE g^−1^ (FW) Condensed tannins in peel: 14.7 mg CE g^−1^ (FW) and seeds: 15.5 mg CE g^−1^ (FW) Main phenolic compounds in peel (DM): Delphinidin-3-O-glucoside: 2032–2644 µg L^−1^ Delphinidin rutinoside: 3255–4407 µg L^−1^ Quercetin-3-glucoside: 2048–2654 µg L^−1^ Valoneic acid dilactone: 103–1430 µg L^−1^ Sinensetin: 1769–4164 µg L^−1^ Rutin: 3608–4055 µg L^−1^ Main phenolic compounds in seed (DM): Cyanidin-3-O-glucoside: 174–345 µg L^−1^ Valoneic acid dilactone: 5947–13,127.81 µg L^−1^ 6-Malonyldaidzin: 67–143 µg L^−1^ Myricitrin: 25–74 µg L^−1^ Gallic acid: 28–71 µg L^−1^	Peel and seeds of ripe, semi-ripe, and ripe fruits of *Citrus reticulata* Blanco.	[140]
	64 volatile compounds detected (DM): Tomato branches had β-carotene (37.23 mg/kg^−1^) and lycopene (3.08 mg/kg^−1^) concentrations. Phenolic compounds in rotten fruit, tomato branches, and green tomato were present at 27.54, 27.09 and 9.90 mg GAE/g, respectively.	Rotten and green tomato fruit and branches of tomato plant.	[141]
	Compounds present (mg/100 g DM): 3-Caffeoylquinic acid: 8.3 - 104 5-Caffeoylquinic acid: 167 - 385 Caffeine: 194–391 Caffeic acid: 3.7 4,5-Dicaffeoylquinic acid: 3.6 - 11.0 1,5-Dicaffeoylquinic acid: 2.3 3,4-Dicaffeoylquinic acid: 0.6 - 7.0	Coffee grounds	[142]
	Compounds (µg/g DM): Gallic acid: 0.72–60.22 Chlorogenic acid: 19.64–337 Caffeic acid: 1.19–6.15	Coffee husks	[143]
	Compounds present in dry matter (%): Crude protein (11.53), crude fiber (1.00), crude fat (15.00), carbohydrate (58.39), pectin (18.2)	Watermelon rind	[144]
	Major compounds (mg/100 g DM): Caftaric acid: 22.4 Viferin: 10.4 Procyanidin B2: 24.6 Quercetin-β-D-glucoside: 288.9	Grape skin	[145]
	Total phenols: 309.14–666.41 mg GAE/100 g DM Total flavonoids: 74.75–120.47 mg QE/100 g DM Total anthocyanins: 8.39–8.95 mg CGE/100 g DM Vitamin C: 68.40–108.04 mg/100 g DM	Peach waste	[146]
	Concentrations of phenolic compounds (mg/100 g DM): Caffeic acid: 57.88 Caffeic acid derivative: 6.41 Chlorogenic acid derivative: 454.34 *p*-Coumaric acid: 7.23 Quercetin derivative: 11.32 Kaempferol derivative: 1.88 Fatty acids (%): Palmitic (C16:0): 29.05–35.60 Stearic (C18:0): 6.71–11.96 Oleic (C18:1-9*c*): 14.59–22.25 Linoleic (C18:2-9,12*c*; *w-6*): 35.62–41.33	Tomato peel	[147]
	Compounds in g/kg^−1^ DM: Chlorophylls: <0.03 Polyphenols: <1.0 Carotenoids: <0.07		[148]
	Lycopene: 9068–17532 mg/kg DM Lycopene: 272 mg/100 g DM		[149] [150]
	Lycopene: 33.83–135 mg/100 g DM Total carotenoids: 65.09–160.04 mg/100 g DM	Guava powder	[151]
	Pectin yield in dry matter (%): Lemon: 10.11 Mandarin: 11.29 Kiwi: 17.30	Peel of lemon, mandarin, and kiwi.	[152]
Biocompounds presents in flowers and flower waste.	Total carotenoids: 0.129–0.173 mg/g FW Total chlorophyll-a: 1.02 mg/g FW Total chlorophyll-b: 0.315 mg/g FW Phenolic content: 130.10–202.30mg GAE/100g FW	Younger and mature leaf of Hibiscus sabdariffa var. sabdariffa.	[153]
	Total phenols: 5.65 μg GAE/mL (DM) Total flavonoids: 0.43 μg QE/mL (DM)	Flower of *Crotalaria juncea*	[154]
	Identification of 42 phenolic compounds, some of them were the following (DM): Acid gallic: 13.402–54.318 (mgGAE/gE) HHDP digalloyl hexose: 4.907–11.884 (mgGAE/gE) Flavogallonic acid: 2.810–6.891 (mgGAE/gE) Ellagic acid: 6.591–67.784 (mgGAE/gE) Kaempferol-3-O-β-d-galactopyranoside: 7.124–29.525 (mg HypE/gE) Quercetin: 3.859–16.758 (mg HypE/gE) Kaempferol: 4.751–20.206 (mg HypE/gE)	Rose blossom (flower)	[155]
	Major compounds (DM): Total phenols: 167.23 mg/g Total flavonoids: 76.11 mg/g Chlorogenic acid: 3.36 mg/g Catechin: 5.21 mg/g Quercetin: 11.01 mg/g	Flowering shoots of *Scrophularia striata Boiss*	[156]
	Yield (% per gram of DM): Deep pink (Portulaca grandiflora): 0.73–1.65 Red (*Rosa ards rovar*): 3.59–6.79 Light red (*Celosia argentea ver*. *cristia*): 0.67–1.43 Orange (*Periskia bleo*): 1.19–3.3 Bluish green (*Alternanthera ficoidea*): 1.1–2.67	Petals Petals Comb of roster Petals Leaves	[157]

FW: fresh weight; DM: dry matter; GAE; gallic acid equivalents, mg gallic acid equivalents/g extract; (mgGAE/gE), mg hyperoside equivalents/g extract; (mg HypE/gE), CE; catechin equivalents, QE; quercetin equivalents, CGE; cyanindin-3-glucoside equivalents.

## 6. Perspectives

A deep study of the bioactive compounds derived from organic waste from the food and agricultural industry is necessary for their reincorporation into other production chains. Those organic residues that are difficult to treat or dispose of, which regularly become an environmental problem, should be incorporated into the circular economy as a priority. Bioactive compounds extracted from agricultural debris are being studied for their capacity to maintain and increase food quality and health benefits. In the case of organic garbage generated in floriculture, a new opportunity and source of bioactive compounds have drawn the attention of the scientific and industrial community; however, a deeper understanding of extraction methods is relevant for obtaining adequate amounts of extracted compounds, energy efficiency, and the least possible environmental impact.

The incorrect separation, use, and exploitation of organic waste reduce the possibilities for the revaluation and extraction of bioactive compounds. The knowledge and ecological attitudes necessary for the reuse of all organic waste has yet to be achieved. In addition, environmentally friendly extraction strategies should be encouraged over traditional extraction methods that use highly polluting solvents. For instance, using supercritical fluids, especially carbon dioxide, is a promising alternative as there are no solvent residuals. However, this extraction method exhibits high energy consumption, but incorporating alternative renewable energy sources (e.g., solar) or applying energy integration strategies could help reduce this inconvenience. In addition, functionality and toxicity tests are crucial, as the extractions will be obtained from waste. More information and more studies are needed to incorporate residues into value chains to stop considering waste as garbage and take advantage of their bioactive compounds for the pharmaceutical, food, agricultural, farming, and aquacultural industries and sectors.

## 7. Conclusions

This review describes various types of waste related to the agricultural, horticultural, and floricultural sectors to obtain compounds with biological activity and their possible applications for reincorporation into productive sectors. Many bioactive compounds are present in these residues, including phenolics, flavonoids, organic acids, volatile compounds, and pigments, that can be reincorporated into different productive sectors, contributing to the circular economy and maximum utilization of natural resources. There are areas of opportunity for the generation of added-value products by incorporating these compounds. For instance, their biological properties (e.g., antioxidant activity) are attractive to the pharmaceutical, cosmetic, and food industries. Finally, their antimicrobial activity needs to be better explored, representing a potential opportunity for using, reincorporating, and developing value-added products, such as gels, films, and bio-insecticides.

## Data Availability

Data are contained within this article.
